# A New Chalcone and Antimicrobial Chemical Constituents of *Dracaena stedneuri*

**DOI:** 10.3390/ph15060725

**Published:** 2022-06-07

**Authors:** Cédric M. Mouzié, Michel-Gael F. Guefack, Boris Y. Kianfé, Héritier U. Serondo, Beaudelaire K. Ponou, Xavier Siwe-Noundou, Rémy B. Teponno, Rui W. M. Krause, Victor Kuete, Léon A. Tapondjou

**Affiliations:** 1 Department of Chemistry, Faculty of Science, University of Dschang, Dschang P.O. Box 67, Cameroon; cedricmouzie@yahoo.com (C.M.M.); kianfeyottaboris@gmail.com (B.Y.K.); serondo1084@gmail.com (H.U.S.); beaudelaireponou@yahoo.fr (B.K.P.); tapondjou2001@yahoo.fr (L.A.T.); 2 Department of Biochemistry, Faculty of Science, University of Dschang, Dschang P.O. Box 67, Cameroon; michelfofack@gmail.com (M.-G.F.G.); kuetevictor@yahoo.fr (V.K.); 3 Higher Pedagogical Institute of Bukavu, Bukavu P.O. Box 854, Democratic Republic of the Congo; 4 Pharmaceutical Sciences Department, School of Pharmacy, Sefako Makgatho Health Sciences University, Pretoria 0204, South Africa; 5 Department of Chemistry, Faculty of Science, Rhodes University, Gahamstown 6139, South Africa; r.krause@ru.ac.za

**Keywords:** *Dracaena steudneri*, dracaenaceae, chalcone, antimicrobial activity

## Abstract

Microbial infections are leading causes of death and morbidity all over the world due to the development of the resistance to antibiotics by certain microorganisms. In this study, the chemical exploration of the ethanol (EtOH) extract of the aerial part of *Dracaena stedneuri* (Dracaenaceae) led to the isolation of one previously unreported chalcone derivative, i.e., 2′,4′-dihydroxy-2,3′-dimethoxychalcone (**1**), together with 12 known compounds: 8-(*C*)-methylquercetagetin-3,6,3′-trimethyl ether (**2**), methylgalangine (**3**), quercetin (**4**), kaempferol (**5**), 6,8-dimethylchrysin (**6**), ombuine-3-*O*-rutinoside (4ʹ,7-dimethylquercetin-3-*O*-*α*-L-rhamnopyranosyl-(1 → 6) -*β*-D-glucopyranoside) (**7**), alliospiroside A (**8**), *β*-sitosterol 3-*O*-glucopyranoside (**9**), ishigoside (**10**), betulinic acid (**11**), oleanolic acid (**12**), and lupeol (**13**). The structures were determined by spectroscopic and spectrometric analysis including 1- and 2-Dimensional Nuclear Magnetic Resonance (1D- and 2D-NMR), High-Resolution Electrospray Ionization Mass Spectrometry (HRESIMS), and comparison with literature data. The isolated secondary metabolites and crude extract displayed antibacterial activity against some multidrug-resistant strains with minimal inhibitory concentration (MIC) values ranging from 32 to 256 μg/mL. The antibacterial activity of compound **13** against *Enterobacter aerogenes* ATCC13048 (MIC value: 32 μg/mL) was higher than that of chloramphenicol used as the reference drug (MIC = 64 μg/mL).

## 1. Introduction

**Figure 1:**
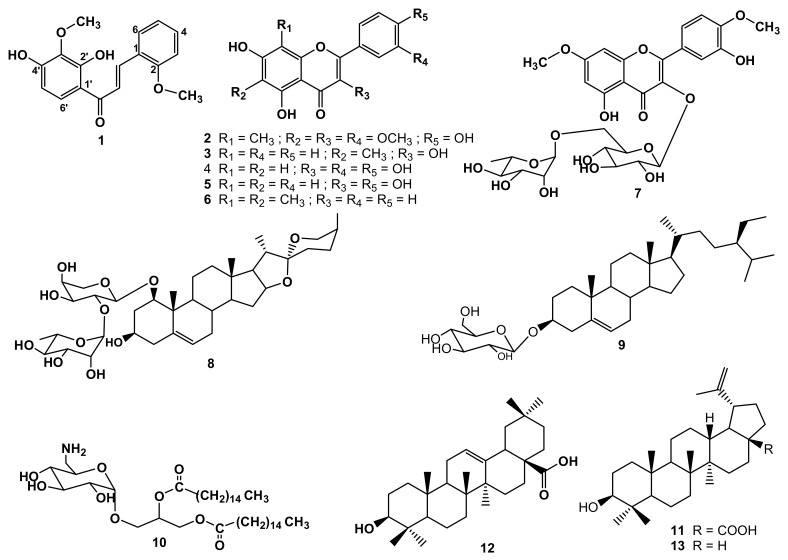
Structures of secondary metabolites obtained from *D. steudneri* (**1**–**13**).

Antibiotics are used in the treatment of infectious diseases, which still represent an important source of mortality in the world. However, the broad and incorrect uses of antimicrobial agents led to the development of microbial resistance [1]. It is therefore necessary to find new effective solutions to combat these multidrug-resistant (MDR) microorganisms. Plant extracts and isolated compounds could constitute an alternative solution to this problem because some of them are used in traditional medicine to treat several ailments, including microbial infections. The genus *Dracaena* (Dracaenaceae) contains approximately 100 species of shrubs and trees spread in tropical and subtropical regions of the world [2,3]. *Dracaena steudneri* Engl. is distributed in the DR Congo, Ethiopia, and East to southern African countries. It is an evergreen tree of about 5 m in height with pale white-yellow-green flowers and green fruits [4,5]. The extract from this plant is used in traditional medicine in Tanzania to cure splenomegaly, hernias, asthma, and chest problems [6], and in Rwanda to treat liver diseases [7]. In Kenya, the extract from the stem is used to manage hepatic liver ailments, is a cure for measles, and reduces pain during childbirth [8,9]. The previous phytochemical study of its leaves led to the discovery of six new flavonoids together with thirteen known congeners [9]. As part of our research program based on the exploration of bioactive secondary metabolites from Dracaenaceae [10,11,12,13], we describe in the present paper the isolation and structure elucidation of thirteen compounds (Figure 1) including a new chalcone derivative from the EtOH extract of *D. steudneri.* Furthermore, the EtOH extract, the ethyl acetate, *n*-butanol soluble fractions, and some isolated metabolites were screened for their antibacterial activity against a panel of MDR bacteria.

## 2. Results and Discussion

The phytochemical investigation of *D. steudneri* led to the discovery of a new chalcone derivative: 2′,4′-dihydroxy-2,3′-dimethoxychalcone (**1**), together with twelve known compounds. The known compounds were identified as: 8-*C*-methylquercetagetin-3,6,3′-trimethyl ether (**2**) [14], methylgalangine (**3**) [15], quercetin (**4**) [16], kaempferol (**5**) [17], 6,8-dimethylchrysin (**6**) [18], ombuine-3-*O*-rutinoside (4′,7-dimethylquercetin-3-*O*-*α*-L-rhamnopyranosyl-(1 → 6)-*β*-D -glucopyranoside) (**7**) [19], alliospiroside A (**8**) [20], *β*-sitosterol 3-*O*-glucopyranoside (**9**) [21], ishigoside (1,2-(dipalmitoyl)-3-*O*-*α*-*D*-(6-desoxy-6-amino-glucopyranosyl)glycerol) (**10**) [22], betulinic acid (**11**) [23], oleanolic acid (**12**) [24], and lupeol (**13**) [24] (Figure 1).

The HRESIMS of compound **1** obtained as a yellow powder, presented a sodium adduct at *m*/*z* 323.0887 [M+Na]^+^ (calcd. for C_17_H_16_O_5_Na^+^: 323.0895). Its ^1^H-NMR spectrum revealed two aromatic proton resonances at *δ*_H_ 7.55 (d, 1H, *J* = 8.8 Hz, H-6′) and 6.48 (d, 1H, *J* = 8.9 Hz, H-5′). Resonances of four aromatic protons were also depicted at *δ*_H_ 7.55 (brd, 1H, *J* = 8.8 Hz, H-6); 7.32 (ddd, 1H, *J* = 8.7, 7.4, 1.7 Hz, H-4); 6.93 (td, 1H, *J* = 7.5, 1.0 Hz, H-5); and 6.88 (dd, 1H, *J* = 8.4, 1.0 Hz, H-3) suggesting the presence of one *ortho* disubstituted aromatic ring [9]. It also exhibited signals of two *trans*-oriented olefinic protons at *δ*_H_ 8.12 (d, 1H, *J* = 15.6 Hz, H-*β*) and 7.62 (d, 1H, *J* = 15.6 Hz, H-*α*) characteristic of chalcones [25,26]. The proton resonances observed at *δ*_H_ 3.95 (s, 3H, 3′-OMe) and *δ*_H_ 3.86 (s, 3H, 2-OMe) evidenced the existence of two methoxyl groups (Table 1 and Figure S1,Figure S2,Figure S3,Figure S4,Figure S5,Figure S6).

The ^13^C-NMR spectrum displayed seventeen resonances including a carbonyl at δ_C_ 193.2 and an *α*,*β*-unsaturated system with resonances depicted at *δ*_C_ 120.9 (C-*α*) and 140.3 (C-*β*), characteristic of a chalcone skeleton [25,27]. The signals of two methoxyl groups were observed at *δ*_C_ 60.8 (3′-OCH_3_) and 55.6 (2-OCH_3_). The ^1^H and ^13^C signals were totally assigned by inspection of the Correlation Spectroscopy (^1^H-^1^H COSY) spectrum combined with data from Heteronuclear Single Quantum Coherence (HSQC) and Heteronuclear Multiple Bond Correlation (HMBC) spectra (Table 1). The location of the methoxyl groups was deduced from the strong HMBC correlations observed between the protons at δ_H_ 3.95 and 3.86, and the aromatic carbons at *δ*_C_ 134.3 (C-3′) and δ_C_ 159.0 (C-2), respectively. This was further confirmed by the Nuclear Overhauser Effect Spectroscopy (NOESY) correlation depicted between the protons at *δ*_H_ 3.86 (2-OCH_3_) and the proton at *δ*_H_ 6.88 (H-3). The substitution pattern of the B ring was previously identified in some flavonoids, namely (2*R*)-7-hydroxy-2′,8-dimethoxyflavanone [9] as well as irisones A and B [28]. Hence, compound **1** was characterized as a previously unreported chalcone named 2′,4′-dihydroxy-2,3′-dimethoxychalcone.

The crude extract, fractions, and some isolated compounds were evaluated for their antibacterial activity using the broth microdilution method. The cut-off values of the minimum inhibitory concentrations (MIC) classification scale, indicating the antibacterial activity of extracts and secondary metabolites derived from plants, are well known [29]. According to this scale, the antimicrobial activity of plant extracts can be classified as significant (MIC vakue < 100 µg/mL), moderate (100 < MIC value ≤ 625 µg/mL), and weak (MIC value > 625 µg/mL), while that of pure compounds can be classified as significant (MIC < 10 µg/mL), moderate (10 < MIC value ≤ 100 µg/mL), and weak (MIC value > 100 µg/mL). Consequently, a significant antibacterial activity (MIC < 100 μg/mL) was observed for the crude EtOH extract of *D. steudneri* against *Enterobacter aerogenes* ATCC13048 (Table 2). However, a moderate inhibition (10 < MIC value ≤ 100 μg/mL) was observed for methylgalangine (**3**), quercetin (**4**), kaempferol (**5**), 6,8-dimethylchrysin (**6**), *β*-sitosterol 3-*O*-glucopyranoside (**9**), and lupeol (**13**) against different bacterial strains used (*Escherichia coli*, *Klebsiella pneumoniae*, *Enterobacter aerogenes*, *Providencia stuartii,* and *Pseudomonas aeruginosa*). The results obtained are in agreement with the literature since flavonoids, saponins, and triterpenes are known to exhibit potent antibacterial activity [29,30,31]. Among the tested compounds, lupeol (**13**) was the most active with an MIC value of 32 μg/mL against *Enterobacter aerogenes*. The reference antibiotic chloramphenicol inhibited the growth of all studied bacteria and its activity against some microbial strains which were as good as those of lupeol (**13**). Although the most active compounds exhibited moderate antibacterial activity, it should be noted that the recorded MIC values were close to those of the reference drug on the corresponding efflux pump-expressing MDR bacteria. This clearly suggests that a possible combination with other antibiotics or efflux pump inhibitors should be envisaged to improve their potency. The lack of inhibition by some of the tested compounds could be explained by the multi-resistant patterns of the bacterial strains used. Unlike Gram-positive bacteria, the Gram-negative bacteria used in this work are characterised by the multi-drug resistance phenotype, with the main mechanism of resistance being the extracellular expulsion of drugs via active efflux. In these types of bacteria, efflux pumps play many roles, among which are the reduction of the intracellular concentration of exogenous substances such as antibiotics [32,33]. Furthermore, it was reported by Epang et al. (2016) that Gram-negative bacteria are surrounded by two membranes: the cytoplasmic cell membrane and the outer membrane containing lipopolysaccharides. Gram-positive bacteria instead do not have the additional outer membrane layer but share the commonality of having a cell wall surrounding the cytoplasmic membrane of the cell wall consisting of peptidoglycan. Gram-negative bacteria tend to be more resistant to antimicrobial agents than Gram-positive bacteria, because of the presence of the additional protection afforded by the outer membrane [34].

The antimicrobial activity of quercetin (**4**), *β*-sitosterol 3-*O*-glucopyranoside (**9**), oleanolic acid (**12**), and lupeol (**13**) have already been evaluated. Quercetin exhibited a moderate activity against *Candida albicans* with an MIC value of 32 µg/mL [35], while it was shown that oleanolic acid possesses a broad range of antibacterial activity, mainly against Gram-positive bacteria [30]. Lupeol was reported to display significant zones of inhibition in the cultures of 18 hospital strains of the Gram-negative bacteria *Pseudomonas aeruginosa* and *Klebsiella pneumoniae* at a concentration of 30 μg/100 μL [36]. Some of the isolated compounds were also tested against three strains of *Staphylococcus aureus* (Gram-positive bacteria). As shown in Table S1 (Supplementary data), methylgalangine (**3**), quercetin (**4**), kaempferol (**5**), *β*-sitosterol 3-*O*-glucopyranoside (**9**), and betulinic acid (**11**) exhibited significant activity against *S. aureus* ATCC25923 with MIC values ranging from 4 to 8 µg/mL. A significant activity was also observed for compounds **3**, **5**, and **6** against *S. aureus* MRSA3 as well as for compounds **3**, **5**, and **9** against *S. aureus* MRSA6. Our results further confirmed the fact that Gram-negative bacteria are more resistant to antimicrobial agents than Gram-positive bacteria [34].

## 3. Materials and Methods

### 3.1. General Experimental Procedures

HRESIMS spectra were recorded on a Bruker Daltonics Compact Quadrupole Time of Flight (QToF) Mass Spectrometer using an electrospray ionisation probe, using direct injection in the positive mode. A Bruker Advanced III 400 MHz (400.13 MHz; 100.62 MHz) spectrometer at 25 °C was used to record ^1^H-, ^13^C-NMR, and 2D NMR spectra. All chemical shifts (*δ*) are given in ppm with reference to the residual solvent signal and coupling constants (*J*) are in Hz. Column chromatography was performed on Sephadex LH-20 and silica gel 60 (0.040–0.063 mm, Merck). TLC was performed on percolated silica gel 60 F_254_ (Merck) plates developed with Hexane-CH_2_Cl_2_, Hexane-EtOAc, CH_2_Cl_2_, CH_2_Cl_2_-MeOH, and EtOAc-MeOH. Thin Layer Chromatography (TLC) spots were visualized under UV light (254 and 365 nm) and by spraying with 10% aqueous or methanolic H_2_SO_4_ followed by heating at 90 °C.

### 3.2. Plant Material

The leaves of *D. steudneri* were collected in Sud-Kivu, an East region of the DR Congo in September 2019. The specimen was identified at the Research Centre in Natural Sciences Lwiro (CRSN/Lwiro), where a voucher specimen (No. 375) was deposited.

### 3.3. Extraction and Isolation

The air-dried and ground leaves of *D. steudneri* (4 kg) were extracted three times (each time for 24 h) with EtOH (96%, 18 L) by maceration. The solvent was evaporated to yield 554.3 g of crude extract. An amount of 544.3 g of this extract was suspended in distilled water (600 mL) and consecutively extracted with EtOAc and *n*-BuOH to give, after evaporation of the solvent, 102.8 g and 114.5 of EtOAc and *n*-BuOH fractions, respectively. A portion of the EtOAc fraction (98 g) was subjected to silica gel column chromatography eluting with gradients of *n*-hexane/EtOAc (90:10 → 10:90) and EtOAc/MeOH (95:5 → 70:30) as mobile phases to afford eight main sub-fractions (Fr.1–Fr.8). Fr.3 (6.3 g) was subjected to Sephadex LH-20 column chromatography using MeOH as the eluent to give four sub-fractions (Fr.3-1–Fr.3-4). Betulinic acid (**11**) (15 mg) was obtained from the sub-fraction Fr.3-1 (1.4 g) by recrystallization in *n*-hexane/EtOAc (80:20) followed by simple filtration. To remove chlorophylls, sub-fractions Fr.3-2, Fr.3-3, and Fr.3-4 were separately submitted to Sephadex LH-20 column chromatography eluted with *n*-hexane/CH_2_Cl_2_/MeOH (7:4:0.5) to afford sub-fractions Fr.3-2-1 (620.2 mg), Fr.3-3-1 (350 mg), and Fr.3-4-1 (28.5 mg), respectively. Fr.3-2-1 was then submitted to silica gel column chromatography, eluted with *n*-hexane/EtOAc (90:10) to yield compounds **6** (25.2 mg) and **12** (62.1 mg), while Fr.3-3-1 and Fr.3-4-1 were purified by silica gel column chromatography using *n*-hexane/CH_2_Cl_2_ (1:1) to give compounds **1** (7 mg) and **3** (8 mg), respectively. The sub-fraction Fr.4 (14 g) was chromatographed on Sephadex LH-20 column eluted with MeOH to yield a mixture that was further separated on a silica gel column using *n*-hexane/EtOAc (80:20) as the eluent to yield two sub-fractions: Fr.4-1 (103.4 mg) and Fr.4-2 (630 mg). The sub-fraction (Fr.4-2) was first submitted to Sephadex LH-20 column chromatography eluted with *n*-hexane/CH_2_Cl_2_/MeOH (7:4:0.5), then to a silica gel column chromatography eluted with *n*-hexane/EtOAc (80:20) to give compounds **2** (15 mg) and **5** (12 mg). Fr.5 (61.6 g) was subjected to Sephadex LH-20 column chromatography eluted with MeOH to give three the sub-fractions Fr.5-1–5-3. The purification of Fr.5-3 (122.4 mg) by column chromatography using silica gel eluting with a mixture of *n*-hexane/EtOAc (70:30) gave compounds **4** (11 mg) and **5** (5 mg). The recrystallization of Fr.2 (12.6 g) in *n*-hexane/EtOAc, 90:10 yielded compound **13** (60 mg). Part of the *n*-BuOH extract (108 g) was fractionated by silica gel column chromatography eluted with EtOAc with increasing amounts of MeOH to give five main sub-fractions A-E. Sub-fraction D (34.2 g) was chromatographed on a silica gel column using EtOAc/MeOH/H_2_O (95:5:2) as the eluent to afford four sub-fractions (D_1_-D_4_). Recrystallization and filtration of the sub-fraction D_2_ (255 mg) yielded *β*-sitosterol 3-*O*-glucopyranoside (**9**) (12 mg) while sub-fraction D_4_ (22.4 g) was repeatedly purified using silica gel column chromatography eluted with EtOAc/MeOH/H_2_O (90:10:5) and EtOAc/MeOH/H_2_O (95:5:2) to give compounds **7** (58.5 mg), **8** (13.5 mg), and **10** (8 mg).

***2′,4′-dihydroxy-2,3′-dimethoxychalcone*** (**1**)**:** Yellow amorphous powder; HRESIMS: *m*/*z* 323.0887 [M+Na]^+^ (Calcd. for C_17_H_16_O_5_Na^+^: 323.0895). ^1^H-NMR (CDCl_3_, 400 MHz and ^13^C-NMR (CDCl_3_, 100 MHz) data: see Table 1.

### 3.4. Antimicrobial Activity

The MIC and MBC of the tested bacteria were determined by the broth microdilution INT colorimetric assay as previously described [37,38]. The tested samples (plant extract, fractions, some of the isolated compounds, and chloramphenicol) were dissolved in DMSO/MHB. The final concentration of DMSO in the sample solution was less than 2.5%, a concentration innocuous to bacterial growth [39,40]. The solution obtained was then added to MHB and a series of two-fold dilutions were performed. Afterward, prepared inoculum (1.5 × 10^6^ CFU/mL) was added. The microplates were sealed and incubated aerobically for 18 h at 37 °C. Wells containing DMSO and inoculum were used as negative controls, whereas those containing chloramphenicol were used as positive controls, for Gram-negative bacteria, including *Escherichia coli* ATTC10536 and AG102, *Enterobacter aerogenes* ATCC13048, *Klebsiella pneumoniae* ATCC11296 and KP55, *Providencia stuartii* PS2636, and *Pseudomonas aeruginosa* PA01. After the incubation period, 40 mL of INT (0.2 mg/mL) was added to each well and re-incubated for 30 min. The MIC value was defined as the lowest sample concentration that did not induce a colour change of the medium, thereby exhibiting complete inhibition of bacterial growth. The MBC was determined by adding 50 µL aliquots of the preparations, which did not show any bacterial growth after incubation during the MIC assays, to 150 µL of MHB. These preparations were incubated at 37 °C for 48 h. The MBC was considered as the lowest sample concentration that did not produce any colour change of the medium after the addition of INT as mentioned above [41]. All assays were performed in triplicate and repeated thrice.

## 4. Conclusions

The chemical investigation of the EtOH extract of the leaves of *D. steudneri* led to the isolation, characterization, and identification of a new chalcone derivative together with twelve other known compounds including six flavonoids, two saponins, one glyceride glycoside, and three triterpenes. A panel of spectroscopic and spectrometric methods as well as the comparison with published data were used to characterize the isolated compounds. The extract, fractions, and some pure compounds were screened for their antimicrobial activity against several multidrug-resistant bacteria, the results of which are very promising since the MIC values recorded were in many cases close to those of the reference drug chloramphenicol. The study also evidenced the antibacterial potential of flavonoids, saponins, and triterpenes, although a study of their mechanism of action remains to be explored.

## Figures and Tables

**Table 1:** ^13^C- and ^1^H-NMR spectroscopic data of compound **1** (100 and 400 MHz, resp.; CDCl_3_; δ in ppm).

Position	δ_C_, Type	δ_H_ (*J* in Hertz)
C=O	193.2, C	/
α	120.9, CH	7.62, d (15.6)
β	140.3, CH	8.12, d (15.6)
1	123.7, C	/
2	159.0, C	/
3	111.3, CH	6.88, dd (8.4, 1.0)
4	132.0, CH	7.32, ddd (8.8, 7.5, 1.7)
5	120.8, CH	6.93, td (7.6, 1.0)
6	129.6, CH	7.55, brd (8.8)
1′	115.2, C	/
2′	157.7, C	/
3′	134.3, C	/
4′	155.0, C	/
5′	106.3, CH	6.48, d (8.9)
6′	126.3, CH	7.55, d (8.8)
2-OMe	55.6, CH_3_	3.86, s
3′-OMe	60.8, CH_3_	3.95, s

**Table 2:** Antimicrobial activities (MIC and MBC values in μg/mL) of the extract, fractions, isolated compounds, and chloramphenicol.

**Bacterial Strains**		**E1**		**E2**		**E3**		**3**		**4**		**5**		**CHL**	
		**MIC**	**MBC**	**MIC**	**MBC**	**MIC**	**MBC**	**MIC**	**MBC**	**MIC**	**MBC**	**MIC**	**MBC**	**MIC**	**MBC**
*E. coli*	ATTC10536	128	512	128	>256	128	>256	64	>256	128	>256	128	>256	128	16
	AG102	128	-	128	>256	256	>256	64	>256	128	>256	64	>256	64	64
*E. aerogenes*	ATCC13048	64	256	128	>256	128	>256	128	>256	128	>256	64	>256	128	64
*K. pneumoniae*	ATCC11296	128	-	256	>256	128	>256	64	>256	64	256	128	>256	128	64
	KP55	128	-	256	>256	256	>256	128	>256	128	>256	64	>256	64	64
*P. stuartii*	PS2636	128	-	256	>256	128	>256	128	>256	128	>256	64	>256	32	32
*P. aeruginosa*	PA01	128	-	128	>256	512	>256	64	>256	64	>256	128	>256	64	64
**Bacterial Strains**		**6**		**9**		**11**		**12**		**13**			**CHL**		
		**MIC**	**MBC**	**MIC**	**MBC**	**MIC**	**MBC**	**MIC**	**MBC**	**MIC**		**MBC**	**MIC**		**MBC**
*E. coli*	ATTC10536	128	>256	64	>256	128	>256	128	>256	128		>256	16		128
	AG102	256	>256	64	>256	128	>256	256	>256	128		>256	64		64
*E. aerogenes*	ATCC13048	256	>256	128	>256	128	>256	128	>256	32		256	64		128
*K. pneumoniae*	ATCC11296	128	>256	64	>256	256	>256	128	>256	128		>256	64		128
	KP55	64	>256	64	>256	256	>256	256	>256	128		>256	64		64
*P. stuartii*	PS2636	128	>256	128	>256	512	>256	128	>256	128		>256	32		32
*P. aeruginosa*	PA01	128	>256	64	>256	128	>256	512	>256	128		>256	64		64

**MIC:** Minimal Inhibitory Concentration; **MBC:** Minimal Bactericidal Concentration; **3:** methylgalangine; **4:** quercetin; **5:** kaempferol; **6:** 6,8-dimethylchrysin; **9:** *β*-sitosterol 3-*O*-glucopyranoside; **11:** betulinic acid; **12:** oleanolic acid; and **13:** lupeol; E1: crude extract; E2: *n*-butanol fraction; E3: AcOEt fraction; CHL: chloramphenicol used as positive control.

## Data Availability

Not applicable.

## Supplementary Materials

The following supporting information can be downloaded at: https://www.mdpi.com/article/10.3390/ph15060725/s1, Figure S1: HRESI-MS of compound **1**; Figure S2: ^1^H-NMR spectrum of compound **1**; Figure S3: ^13^C NMR spectrum of compound **1**; Figure S4: ^1^H-^1^H COSY spectrum of compound **1**; Figure S5: HSQC spectrum of compound **1**; Figure S6: HMBC spectrum of compound **1**; Table S1: MIC and MBC (in μg/mL) of isolated compounds and chloramphenicol against gram-positive bacterial strains; Table S2: Characteristics of microorganisms used; References [42,43,44,45,46] are cited in the supplementary materials.

## Sample Availability

Samples of some of the isolated compounds are not available from the authors.
