# Supplementary material for: A New Chalcone and Antimicrobial Chemical Constituents of Dracaena stedneuri
Source: Pharmaceuticals (Basel). 2022 Jun 7;15(6):725. doi: 10.3390/ph15060725 (PMC9228254; doi:10.3390/ph15060725)
Supplement: Supplementary file 1 [file pharmaceuticals-15-00725-s001.zip › pharmaceuticals-1624567-supplementary.pdf]

A new chalcone and antimicrobial chemical constituents of *Dracaena stedneuri* Engl.

Cédric M. Mouzié[1], Michel-Gael F. Guefack[2], Boris Y. Kianfé[1], Héritier U. Serondo[1,3], Beaudelaire K. Ponou[1], Xavier Siwe-Noundou[4,*], Rémy B. Teponno[1,*], Rui W. M. Krause[5], Victor Kuete[2] and Léon A. Tapondjou[1]

1 Department of Chemistry, Faculty of Science, University of Dschang, P.O. Box 67, Dschang, Cameroon; cedricmouzie@yahoo.com (C.M.M.); kianfeyottaboris@gmail.com (B.Y.K.); serondo1084@gmail.com (H.U.S.); beaudelaireponou@yahoo.fr (B.K.P.); remyteponno@gmail.com (R.B.T.); tapondjou2001@yahoo.fr (L.A.T.)

2 Department of Biochemistry, Faculty of Science, University of Dschang, P.O. Box 67, Dschang, Cameroon; michelfofack@gmail.com (M.-G.F.G.); kuetevictor@yahoo.fr (V.K.)

3 Higher Pedagogical Institute of Bukavu, P.O. Box 854, Bukavu, DR Congo

4 Pharmaceutical Sciences Department, School of Pharmacy, Sefako Makgatho Health Sciences University, Pretoria, 0204, South Africa; xavier.siwenoundou@smu.ac.za / xavsiw@gmail.com (X.S.-N.)

5 Department of Chemistry, Faculty of Science, Rhodes University, P.O. Box 94, Gahamstown, South Africa; r.krause@ru.ac.za (R.W.M.K.)

* Correspondence: xavier.siwenoundou@smu.ac.za (X.S.-N.); Tel. +27 12 521 5647; remyteponno@gmail.com (R.B.T.); Tel: +237 67 731 2743.

Figure S1. HRESI-MS of compound **1**

Figure S2. ^{1}H-NMR spectrum of compound **1**

Figure S3. ^{13}C NMR spectrum of compound **1**

Figure S4. ^{1}H-^{1}H COSY spectrum of compound **1**

Figure S5. HSQC spectrum of compound **1**

Figure S6. HMBC spectrum of compound **1**

Table S1. MIC and MBC (in µg/mL) of isolated compounds and chloramphenicol against gram-positive bacterial strains.

Table S2. Table S2. Characteristics of microorganisms used

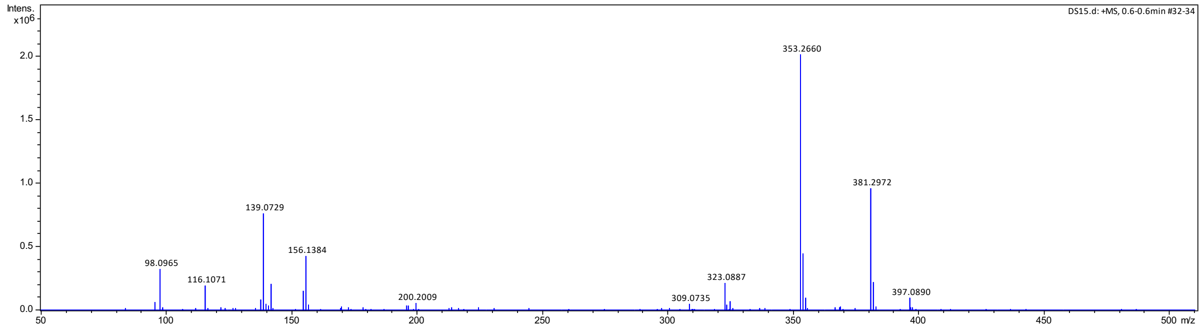

Figure S1. HRESI-MS of compound **1**

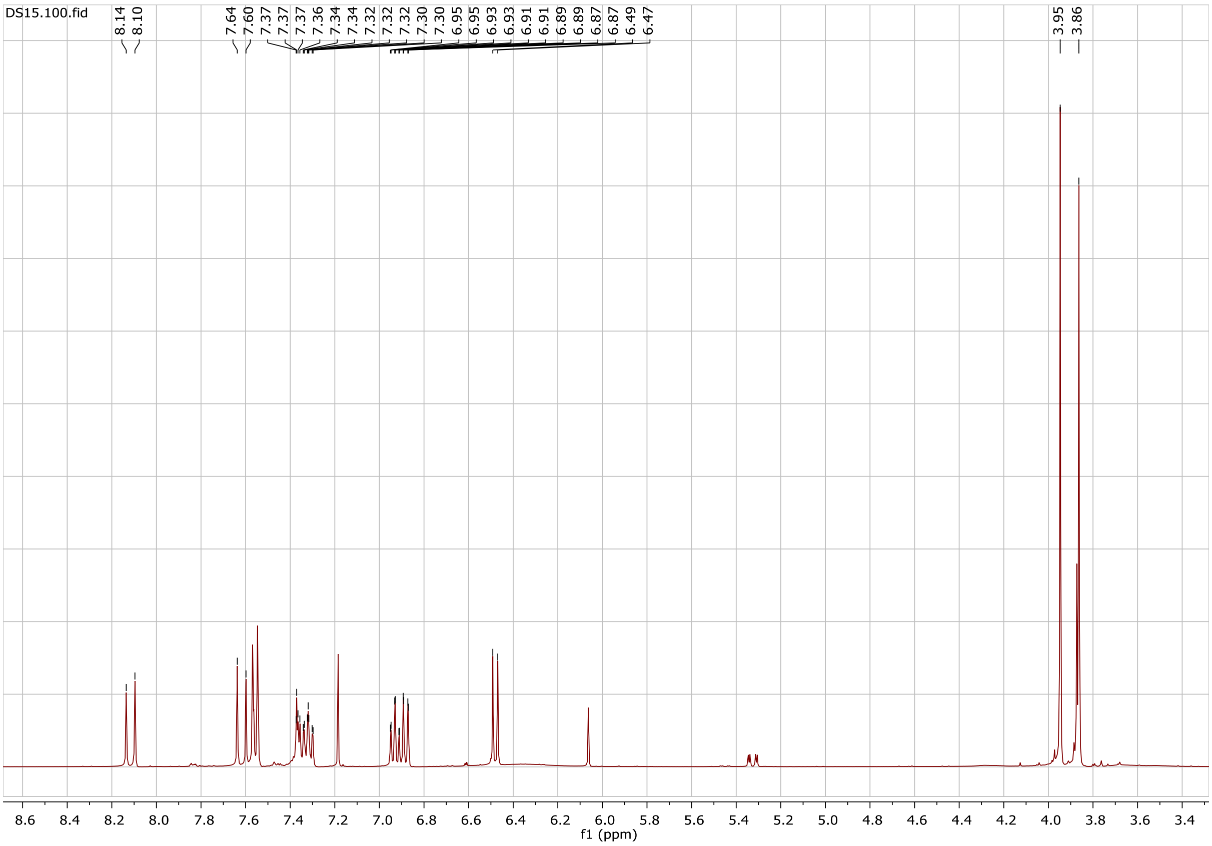

Figure S2. ^{1}H-NMR spectrum of compound **1**

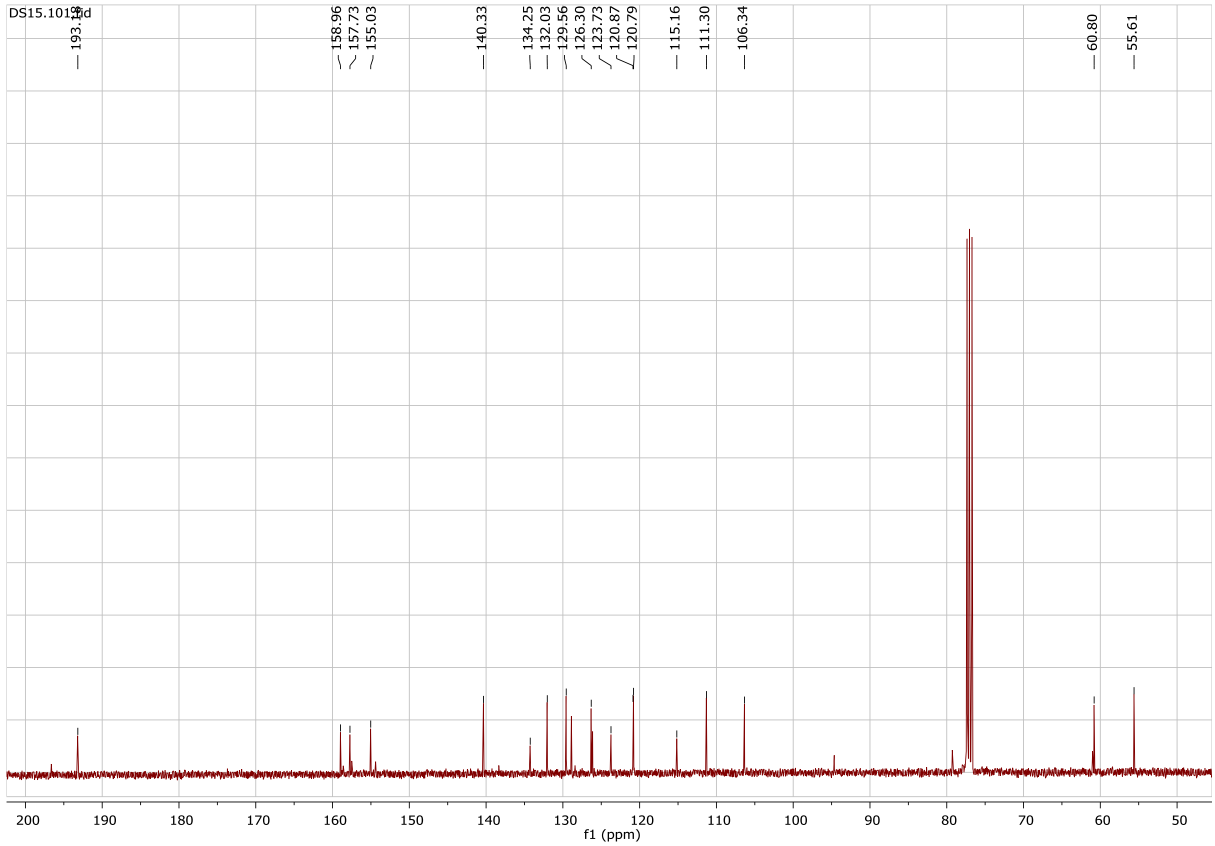

Figure S3. ^{13}C NMR spectrum of compound **1**

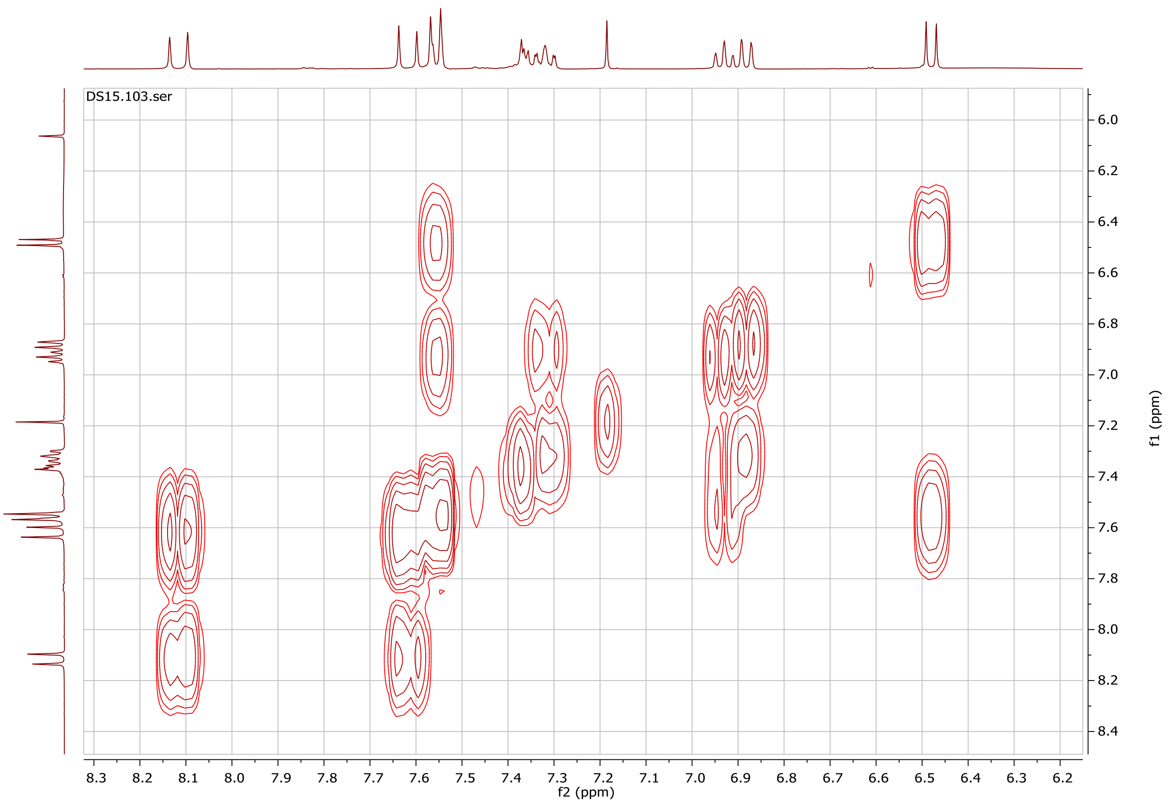

Figure S4. ^{1}H-^{1}H COSY spectrum of compound **1**

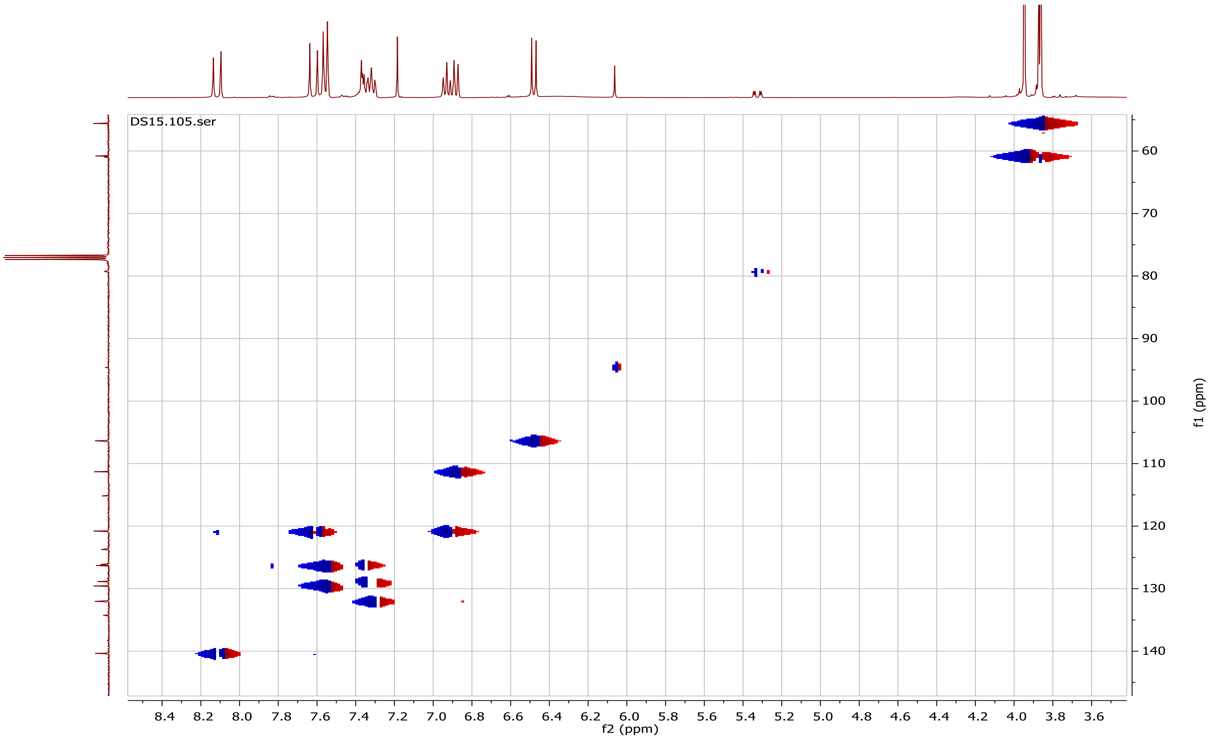

Figure S5. HSQC spectrum of compound **1**

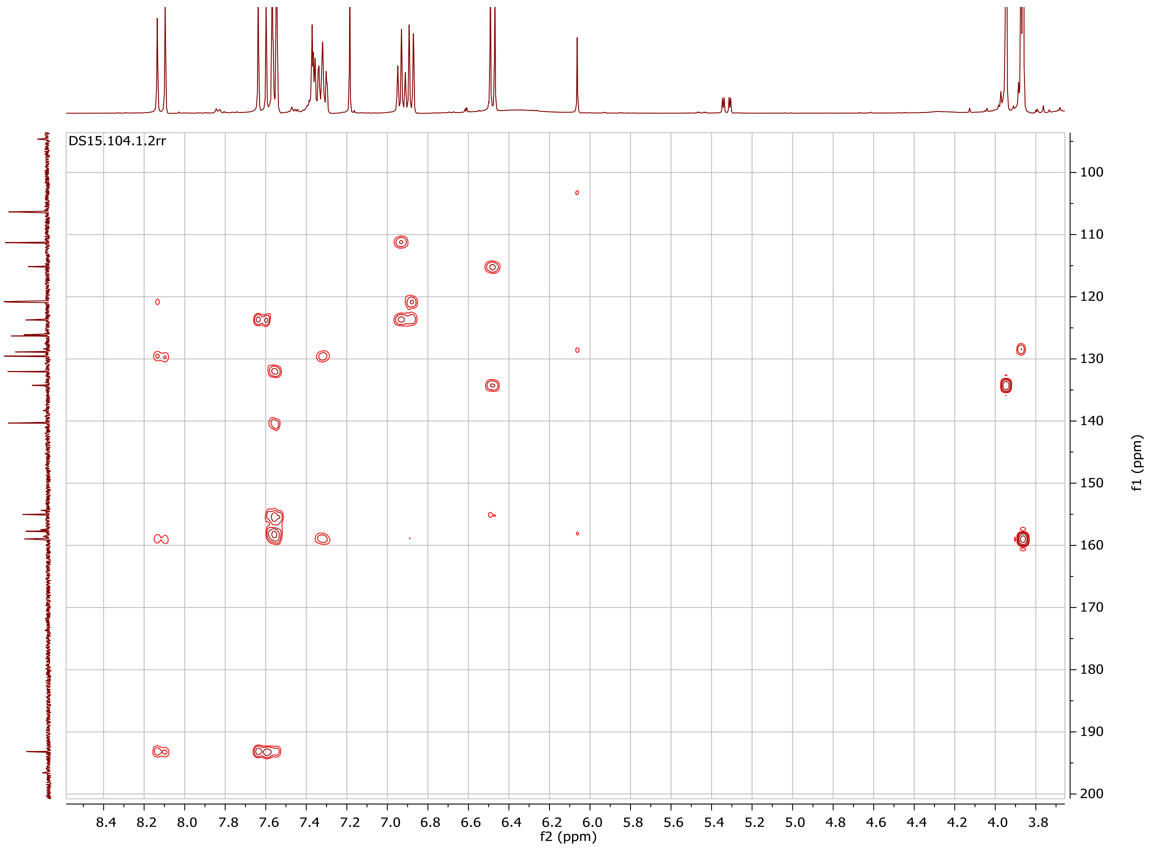

Figure S6. HMBC spectrum of compound **1**

Table S1. MIC and MBC (in µg/mL) of isolated compounds and chloramphenicol against gram-positive bacterial strains.

**Bacterial strains**	**3**		**4**		**5**		**6**		**9**		**11**		**12**		**13**		**CHL**	
	**MIC (MBC)**	**R**	**MIC (MBC)**	**R**	**MIC (MBC)**	**R**	**MIC (MBC)**	**R**	**MIC (MBC)**	**R**	**MIC (MBC)**	**R**	**MIC (MBC)**	**R**	**MIC (MBC)**	**R**	**MIC (MBC)**	**R**
S. aureus																		
**ATCC25923**	8 (64)	8	4 (32)	4	8 (64)	8	16 (64)	4	8 (32)	4	4 (32)	8	16 (64)	4	64 (-)	-	1 (1)	1
**MRSA3**	4 (32)	8	16 (64)	4	8 (32)	4	8 (32)	4	16 (64)	4	16 (64)	4	16 (64)	4	16 (64)	4	1 (4)	4
**MRSA6**	8 (32)	4	16 (64)	4	4 (32)	8	16 (64)	4	8 (32)	4	16 (64)	4	32 (-)	-	64 (-)	-	2 (16)	8

MIC: minimal inhibitory concentration; MBC: minimal bactericidal concentration; R: MBC/MIC ratio (a sample is considered as bacteriostatic or bactericidal when R >4 or ≤4 respectively); (-): MIC or MBC >512 µg/mL.

Table S2. Characteristics of microorganisms used.

Bacteria strains		Characteristics	References
Escherichia coli	**ATTC 10536**	Reference strain	*(Kuete et al., 2010)*
	**AG 102**	AG 100 over expressing *Acr AB* pumps	*(Chevalier et al., 2000)*
Enterobacter aerogenes	**ATCC 13048**	Reference strain	*(Kuete et al., 2010)*
Klebsiella pneumoniae	**ATCC11296**	Reference strain of *Klebsiella pneumoniae*	*Collection of the UMR-MD1 laboratory of the University of the Mediterranean, Marseille, France (Kuete et al., 2010).*
	**Kp 55**	Clinical strain: TET^{r}, AMP^{r}, ATM^{r}, CEF^{r}	
P. stuartii	**PS2636**	*AcrAB-TolcC* associated with porin types OMPF and OMPC	
Pseudomonas aeruginosa	**PA 01**	Reference strain	*Collection of the UMR-MD1 laboratory of the University of the Mediterranean, Marseille, France (Kuete et al., 2010 ; Lorenzi et al., 2009)*
Staphylococcus aureus	**ATCC 25923**	Reference strain	*(Paudel et al., 2012 ; Dzoyem et al., 2013)*
	**MRSA3**	Clinical isolate: Ofxar, Kanr, Tetr, Ermr	
	**MRSA6**	Clinical isolate: Ofxar, Flxr, Kanr, Tetr, Cypr, IM/Csr, Chlr, Genr, Nisr, Ampr	

AMP^{r}, ATM^{r}, CEF^{r}, CHL^{r}, KAN^{r}, NAL^{r}, NOR^{r}, STR^{r} et TET^{r}, resistant to: ampicillin, aztreonam, cefepime, chloramphenicol, kanamycine, nalidixic acid, norfloxine, streptomycine and tetracyclin, respectively; *AcrAB-TolC, MexAB-OprM* : Efflux pomp.

References

Chevalier, J.; Pages, J.M. ; Eyraud, A. ; Mallea, M. Membrane permeability modifications are involved in antibiotic resistance in *Klebsiella pneumoniae*. Biochem. Biophys. Res. Commun. **2000**, 274, 496–499.

Dzoyem, J.P.; Hamamoto, H.; Ngameni, B.; Ngadjui, B.T.; Sekimizu, K. Antimicrobial action mechanism of flavonoids from *Dorstenia* species. Drug Discov. Ther. **2013**, *7*, 66–72.

Kuete, V.; Ngameni,B.; Tangmouo, G.J.; Bola, J.M.; Alibert-Franco, S.; Ngadjui, T.B.; Pages, J.M. Efflux pumps are involved in the defence of gram-negative bacteria against the natural products isobavachalcone and diospyrone. *Antimicrob. Agents Chemother*. **2010**, *54*, 1749–1752.

Lorenzi, V.; Muselli, A.; Bernardini, A.F. ; Berti, L. ; Pages, J.M. ; Amaral, L. ; Bolla, J.M. Geraniol restores antibiotic activities against multidrug-resistant isolates from gram-negative species. Antimicrob. Agents Chemother. **2009,** *53*, 2209–2211.

Paudel, A.; Hamamoto, H.; Kobayashi, Y. ; Yokoshima, S.; Fukuyama, T.; Sekimizu, K. Identification of novel deoxyribofuranosyl indole antimicrobial agents. J. Antibiot. **2012**, *65*, 53–57.
